# Development of a QCM-D-Based Aptasensor for the Real-Time Detection of β-Lactoglobulin

**DOI:** 10.3390/bios15090563

**Published:** 2025-08-27

**Authors:** Tuba Çanak-Ipek, Melis Güler Girbas, Nicolas Casadei, Christian Schlensak, Anna-Kristina Marel, Meltem Avci-Adali

**Affiliations:** 1 Department of Thoracic and Cardiovascular Surgery, University Hospital Tübingen, 72076 Tübingen, Germany; tuba.canak-ipek@uni-tuebingen.de (T.Ç.-I.); melis-gueler.girbas@uni-tuebingen.de (M.G.G.); christian.schlensak@med.uni-tuebingen.de (C.S.); 2 Institute of Medical Genetics and Applied Genomics, University of Tübingen, 72076 Tübingen, Germany; nicolas.casadei@med.uni-tuebingen.de; 3 NGS Competence Center Tübingen, University of Tübingen, 72076 Tübingen, Germany; 4 Department of Food Technology and Bioprocess Engineering, Max Rubner-Institute, Federal Research Institute of Nutrition and Food, 76131 Karlsruhe, Germany; anna-kristina.marel@mri.bund.de

**Keywords:** SELEX, aptamer, β-lactoglobulin, aptasensor, QCM

## Abstract

The prevalence of food allergies has been steadily increasing in recent years. β-lactoglobulin (β-LG), the main allergenic protein of milk and dairy allergies, is more commonly observed in infants and children. In this study, a β-LG-specific aptamer was selected using the combinatorial chemistry process known as systematic evolution of ligands by exponential enrichment (SELEX), and a quartz crystal microbalance with dissipation monitoring (QCM-D)-based aptasensor was developed using a novel surface functionalization technique, which mimics an artificial cell membrane on the QCM-D sensor surface, creating a physiologically relevant environment for the binding of the target to the sensor. Through SELEX combined with next-generation sequencing (NGS), the aptamer Apt 356 was identified. Its binding to β-LG was confirmed via dot blot analysis. The selected Apt 356 was then used for the development of a QCM-D-based sensor. To fabricate the sensor, the quartz surface was functionalized with a supported lipid bilayer (SLB). The β-LG-specific aptamer was immobilized onto this SLB. The results demonstrated that the QCM-D system allows real-time observation and evaluation of the binding of β-LG. While there have been some studies on aptasensors for the β-LG protein, to the best of our knowledge, this is the first QCM-D-based aptasensor developed specifically for β-LG protein detection.

## 1. Introduction

Food allergy is an adverse immune response to specific food proteins, ranging from mild symptoms to life-threatening reactions, making it a significant public health concern [1,2]. Due to their increasing prevalence and severity, food allergies have become a major issue in food safety and public health over the past few decades. Among the most common allergens, cow’s milk particularly affects infants and young children, with β-lactoglobulin (β-LG) identified as the primary protein responsible for allergic reactions [3,4,5]. Symptoms of milk allergy range from mild reactions, such as hives and digestive issues, to severe anaphylaxis. Therefore, effective allergen detection is essential not only for food manufacturers to ensure accurate labeling of allergens, but also for individuals with allergies to manage their dietary intake safely. Given the widespread use of milk in various food products, accurate detection and labeling of milk allergens are crucial for consumer safety.

β-LG is a globular protein belonging to the lipocalin family and is generally found in the milk of most mammals, except for rodents and humans. It has a monomeric molecular weight of approximately 18.3 kDa and naturally occurs in the form of a dimer [6].

Aptamers are short, single-stranded (ss) DNA or RNA molecules, typically 20–90 nucleotides in length, that fold into unique three-dimensional structures and specifically bind to their target molecules. These aptamers are selected through a combinatorial chemistry process known as the systematic evolution of ligands by exponential enrichment (SELEX) [7,8]. Due to their high specificity and affinity, aptamers have significant potential in allergen recognition and biosensor development, leading to the emergence of aptamer-based sensors, or aptasensors [9,10,11].

Quartz crystal microbalance with dissipation monitoring (QCM-D) is a promising technique that enables real-time detection by measuring mass changes on a piezoelectric quartz crystal and analyzing their effects on the system. Functionalizing a QCM-D system with aptamers allows for the development of highly sensitive aptasensors, with detection limits in the nanomolar (nM) range [12].

In this study, a DNA aptamer targeting β-LG was selected using the protein SELEX method. Following the characterization of the selected aptamer, a QCM-D-based aptasensor was developed by immobilizing the β-LG-specific aptamer onto the QCM surface. As surface functionalization, a supported lipid bilayer (SLB) system was chosen to mimic a physiological condition at the sensor interface. The binding events were observed and directly evaluated in real time, demonstrating the aptasensor’s applicability for β-LG detection. Although there are some aptasensor studies for β-LG detection, our study is the first QCM-D-based aptasensor developed specifically for β-LG detection.

## 2. Materials and Methods

### 2.1. Oligonucleotides

An ssDNA library (5′-GCCTGTTGTGAGCCTCCTAAC-49N-CATGCTTATTCTTGTCTCCC-3′) with a centrally randomized region of 49 nucleotides (49N) was used for the initial round of SELEX. During the SELEX, forward (FP: 5′-Biotin-GCCTGTTGTGAGCCTCCTAAC-3′) and reverse (RP: 5′-Phos-GGGAGACAAGAATAAGCATG-3′) primers were used for the polymerase chain reaction (PCR). The 5′-end biotin-labeled Apt 356 (5′-Biotin-GCCTGTTGTGAGCCTCCTAACCTGGTGTGCAAGGTGGACTCTCTTTATCTTGTTGTCAGTCTCATCACCGCATGCTTATTCTTGTCTCCC-3′) was used for the dot blot and QCM measurements. All primers, aptamers, and the start library were HPLC-purified and purchased from Ella Biotech (Fürstenfeldbruck, Germany).

### 2.2. Reagents for the QCM Detection

1-palmitoyl-2-oleoyl-glycero-3-phosphocholine (POPC) and the biotinylated phospholipid 1,2-dipalmitoyl-sn-glycero-3-phosphoethanolamine-N-(cap biotinyl) (sodium salt) (biotin-PE) were obtained from Avanti Polar Lipids (Birmingham, AL, USA). Bovine serum albumin (BSA) and phosphate-buffered saline (PBS) (pH 7.4) containing CaCl_2_ and MgCl_2_ were purchased from Sigma-Aldrich (Taufkirchen, Germany). Streptavidin was obtained from VWR International (Darmstadt, Germany).

The protein β-LG was extracted from whey protein isolate (GermanProt 9000; >90% purity) supplied by Sachsenmilch Leppersdorf GmbH (Leppersdorf, Germany). The isolation procedure followed the method described by Keppler et al. [13], with minor modifications as outlined by Schestkowa et al. [14]. The final β-LG powder exhibited a purity of approximately 96 wt%, as determined by the high-performance liquid chromatography described by Keppler et al. [13].

### 2.3. Performance of Protein SELEX

To perform SELEX, the mixed cellulose esters (MCE) filter membrane (0.45 μm HAWP filter, Merck Millipore, Darmstadt, Germany) was incubated with 0.5 M potassium hydroxide (KOH) to minimize nonspecific binding. The KOH solution was then removed, and the membrane was washed two times with nuclease-free water. The negatively charged membrane was incubated overnight with PBS. Next, 20 µg of purified β-LG (Sigma-Aldrich) in 20 µL PBS was applied to the membrane and incubated for 20 min at room temperature (RT). For the first round of selection, 2 nmol ssDNA start library in 100 µL PBS (pH 4.3) was heated for 5 min at 95 °C and then allowed to cool at RT for 15 min. Subsequently, the start library was applied to the protein-bound membrane, in a total volume of 250 µL PBS (pH 4.3) and incubated at 37 °C for 1 h with gentle shaking. β-LG has an isoelectric point (pI) of approximately 5.2 [15], meaning that at the physiological pH of 7.4, it carries a net negative charge, which could impede the binding of the ssDNA during the initial round of SELEX. Therefore, the pH of the PBS was gradually increased throughout the selection process: pH 4.3 for rounds 1 to 5, pH 5.3 for rounds 6 to 9, pH 6.3 for round 9, and pH 7.4 for rounds 10 to 12.

After the incubation, the membrane was washed with a washing buffer (PBS (pH 4.3 for rounds 1 to 5, pH 5.3 for rounds 6 to 9, pH 6.3 for round 9, and pH 7.4 for rounds 10 to 12) containing 0.2% BSA). The ssDNA bound to the β-LG was then eluted by incubating the membrane twice with 500 µL of 7 M urea in nuclease-free water at 95 °C for 15 min. To remove urea, 500 µL of the solution was transferred into an Amicon Ultra Centrifugal Filter (30 kDa MWCO, Merck Millipore, Darmstadt, Germany) and centrifuged at 14,000× *g* for 10 min. This step was repeated twice, and the purified ssDNA was recovered by placing the filter upside down and centrifuging at 1000× *g* for 2 min. The eluted ssDNA was subsequently amplified via PCR using 1x peqGOLD PCR Master Mix S (VWR Life Science, Radnor, PA, USA), with 0.7 µM forward primer (FP) and 0.7 µM reverse primer (RP), in a total reaction volume of 2 mL. PCR was performed under the following conditions: an initial denaturation at 94 °C for 3 min, followed by 16 cycles of 94 °C for 45 s, 61 °C for 45 s, and 72 °C for 1 min, with a final extension step at 72 °C for 10 min.

Following PCR amplification, the samples were purified using a MinElute PCR purification kit (Qiagen, Hilden, Germany) according to the manufacturer’s instructions. The concentration of the purified double-stranded DNA (dsDNA) was then measured using a spectrophotometer (EON, BioTek Instruments, Winooski, VT, USA).

A total of 40 µg of purified dsDNA was converted to ssDNA using 200 units (5 units/µL) Lambda (λ) exonuclease (New England Biolabs, Hitchin, UK), a 5′→3′ exonuclease that selectively degrades the 5′-phosphorylated strand of DNA. The resulting ssDNA was then purified using the MinElute PCR purification kit (Qiagen, Hilden, Germany), and its concentration was measured spectrophotometrically using a BioTek microplate reader (Agilent Technologies, Santa Clara, CA, USA).

The purified ssDNA was used in the next round of SELEX, and the SELEX process was performed for a total of 12 rounds. Table 1 presents the SELEX conditions for the subsequent selection cycles.

At the end of each selection round, the generated dsDNA and ssDNA were analyzed by gel electrophoresis. Non-denaturing 5% Tris-borate-EDTA (TBE) acrylamide gel electrophoresis was performed to analyze the successful generation of ssDNA, while 10% denaturing urea-polyacrylamide gel was used to identify possible misamplifications or nonspecific bands. Before loading onto the denaturing urea gel, the samples were incubated at 95 °C for 5 min. Electrophoresis was conducted at 100 V for 10 min, followed by 120 V for 30 min. After electrophoresis, the gels were stained with 1x GelRed dye (Biotium, Fremont, CA, USA) for 30 min in 1x TBE buffer.

### 2.4. Quantitative Real-Time PCR (qPCR) for the Monitoring of the Enrichment During SELEX

During the SELEX process, the enrichment of oligonucleotides was analyzed after the 4th, 7th, and 12th rounds by the previously established qPCR method [16]. For this, a standard curve with the SB concentrations of 5, 2.5, 1.25, 0.625, 0.3125, 0.08, and 0.02 pg was used to detect the bound oligonucleotide amount to the target protein β-LG. Therefore, 5 µg of β-LG was immobilized onto an MCE membrane and incubated with the enriched ssDNA (50 pmol) at 37 °C for 30 min under agitation (2 rpm) in a platform shaker with temperature control (Incubator 1000 with Polymax 1040, Heidolph Scientific Products, Schwabach, Germany). Following incubation, the ssDNA bound to the β-LG was eluted by incubating the membrane twice with 500 µL of 7 M urea in nuclease-free water at 95 °C for 15 min. Urea was removed using an Amicon Ultra Centrifugal Filter (30 kDa MWCO, Merck Millipore) as described above.

To detect the bound oligonucleotide amount to the target protein β-LG, a standard curve with the SB concentrations of 5, 2.5, 1.25, 0.625, 0.3125, 0.08, and 0.02 pg was used. The quantitative PCR was performed in a 15 µL reaction mixture containing iQ SYBR Green Supermix (Bio-Rad Laboratories, Feldkirchen, Germany), along with 300 nM of both the forward (5′-GCCTGTTGTGAGCCTCCTAAC-3′) and reverse (5′-GGGAGACAAGAATAAGCATG-3′) primers. The qPCR reactions were performed using a CFX Connect™ Real-Time PCR Detection System (Bio-Rad, Hercules, CA, USA). The thermal cycling conditions included an initial denaturation at 95 °C for 3 min, followed by 30 cycles consisting of denaturation at 95 °C for 45 s, annealing at 58 °C for 20 s, and extension at 72 °C for 20 s. A final extension was carried out at 72 °C for 5 min. At the end of the amplification, a melt-curve analysis was conducted, starting at 70 °C and increasing by 0.5 °C per cycle up to 95 °C.

### 2.5. Next-Generation Sequencing (NGS) and Analysis of the Sequences

In the last SELEX round (round 12), the membrane without the target protein was also incubated with the enriched ssDNA pool to obtain the oligonucleotides unspecifically binding to the membrane (negative selection). Eluted ssDNAs from the negative selection as well as the enriched dsDNA pool against β-LG were amplified by PCR using 1x peqGOLD PCR Master Mix S (VWR Life Science, Radnor, USA), along with OF1 (5′-OF1: GTCTCGTGGGCTCGGAGATGTGTATAAGAGACAGGCCTGTTGTGAGCCTCCTAAC-3′) and 0.7 µM OR1 (5′-TCGTCGGCAGCGTCAGATGTGTATAAGAGACAGGGGAGACAAGAATAAGCATG-3′) in a total reaction volume of 2 mL. The PCR was performed using the following conditions: initial denaturation at 94 °C for 3 min, followed by 6 cycles of 94 °C for 45 s, 61 °C for 45 s, and 72 °C for 1 min, with a final extension step at 72 °C for 10 min (PCR thermocycler, Eppendorf, Hamburg, Germany). The amplicon was purified using the MinElute PCR purification kit (Qiagen, Hilden, Germany) according to the manufacturer’s instructions. Afterwards, the PCR amplicons were indexed using a Nextera adapter (Illumina, San Diego, CA, USA). The resulting libraries were sequenced on the Illumina MiSeq v3 platform (Illumina, San Diego, CA, USA) using 600 cycles chemistry.

To identify the aptamer candidates, a comprehensive analysis of the NGS output was performed, in which multiple sequence alignment and grouping using the MAFFT (Multiple Alignment using Fast Fourier Transform) version 7 platform were applied to classify the sequences. In the final step, we performed a multiple sequence alignment with the selected aptamer sequences using T-Coffee [17]. The secondary structure of the selected aptamers was predicted using RNAfold 2.6.3 via the Vienna RNA Web Services [18,19].

### 2.6. Evaluation of Target Binding Using Dot Blot Assay

To assess the binding of the selected Apt 356 to β-LG, a nitrocellulose membrane (0.45 µm, Thermo Fisher Scientific, Waltham, MA, USA) was pre-soaked in PBS for 10 min. A membrane filter (Thermo Fisher Scientific) was briefly moistened with PBS and placed on the dot blot apparatus (Cleaver Scientific, Rugby, UK), followed by placement of the nitrocellulose membrane. After assembling and closing the device, the membrane was rehydrated by adding 100 µL PBS to each well. The excess buffer was removed by turning on the vacuum. Next, 100 µg β-LG or negative controls, BSA (Sigma-Aldrich) and human serum albumin (HSA, CSL Behring, Marburg, Germany), were immobilized onto the membrane and washed with 100 µL PBS using a vacuum. The membrane was then blocked with 2 mL of blocking solution (PBS containing 5% BSA and 0.01% Tween 20) for 1 h at RT to prevent nonspecific binding. Afterwards, the membrane was incubated with the biotinylated Apt 356 (400 pmol in 700 µL PBS) at 37 °C for 1 h. To remove unbound aptamers, the membrane was washed three times for 10 min each with PBS containing 0.2% BSA. Subsequently, the membrane was incubated for 1 h with streptavidin horseradish peroxidase (HRP) conjugate (Sigma-Aldrich) diluted 1:100 in PBS, followed by four washes with PBS containing 0.01% Tween 20, and a final wash with PBS for 5 min. To detect the aptamer binding, the membrane was treated with ECL Western blotting solution (Sigma-Aldrich) for 5 min, and the membrane was imaged using the iBrightCL1000 system (Thermo Fisher Scientific). Experiments were performed in triplicate.

### 2.7. Quartz Crystal Microbalance with Dissipation Monitoring (QCM-D) Measurements

QCM-D measurements were conducted using a QCell T Q2 system with a dual channel system and a peristaltic pump (3T analytik, Tuttlingen, Germany). Silicon dioxide-coated 10 MHz sensor crystals were purchased from 3T analytik.

The preparation and functionalization of the quartz crystal were performed as previously described [12]. In brief, sensors were cleaned with an alkaline SDS solution, rinsed with Milli Q water and ethanol, and dried under a nitrogen stream. Afterwards, crystals were treated with plasma for 5 min at 100 W using a Zepto HA kHz system (Diener electronics, Ebhausen, Germany). Measurements were conducted at 37 °C with a flow rate of 75 μL min^−1^.

The biotinylated Apt 356 was immobilized on a supported lipid bilayer (SLB) via biotin–streptavidin interaction. The SLB was formed by small unilamellar vesicles composed of biotin-PE/POPC in a ratio of 5/95 at a concentration of 1 mg/mL in TRIS buffer. Streptavidin was dissolved in PBS at a concentration of 100 μg/mL and applied to the SLB. To minimize nonspecific interactions, 1 mg/mL BSA solution was used. The aptamer was dissolved in a TRIS buffer containing 0.1% BSA at a concentration of 0.5 μM. Before adding them to the sensor, the aptamer solutions were heated to 95 °C for 5 min and cooled to RT. β-LG was dissolved in PBS, pH 3.5, at concentrations of 25, 50, 75, 100, 175, 250, and 500 μg/mL. All buffers and reagents were tempered to 37 °C in a water bath before use, and the buffers were degassed in an ultrasonic bath. The incubation steps of streptavidin, Apt 356, and β-LG were conducted without flow to reduce sample consumption. All binding events were observed in real time.

### 2.8. Determination of the Limit of Detection (LOD)

The concentration of β-LG was varied between 25 and 500 μg/mL to establish a calibration curve. The linear regression analysis between the frequency shift and β-LG concentration was used to calculate the LOD according to Equation (1):

(1) $$LOD=\frac{3.3\cdot \sigma_{K}}{S}$$

where $\sigma_{K}$ represents the standard deviation of the intercept, and *S* is the slope of the regression line.

## 3. Results

### 3.1. Enrichment of the Oligonucleotides During the Protein SELEX

**Figure 1:**
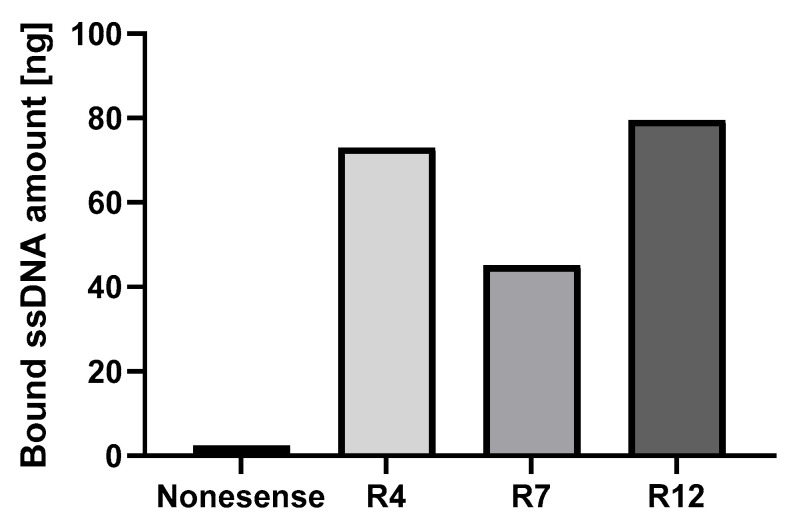
Monitoring of ssDNA enrichment during SELEX using qPCR. A total of 5 µg β-LG was immobilized onto an MCE membrane, and the binding of 50 pmol of the enriched ssDNA aptamer pools from SELEX rounds 4, 7, and 12, as well as the nonsense ssDNA control, was measured using qPCR.

To detect the enrichment of the aptamers, the binding of the selected aptamer pools from rounds 4, 7, and 12 to the β-LG protein was analyzed using qPCR (Figure 1). Compared to the nonsense ssDNA control, the pool from the 12th SELEX round exhibited approximately 32-fold higher binding.

### 3.2. Analysis of the Binding of the Selected Aptamer Using Dot Blot Assay

**Figure 2:**
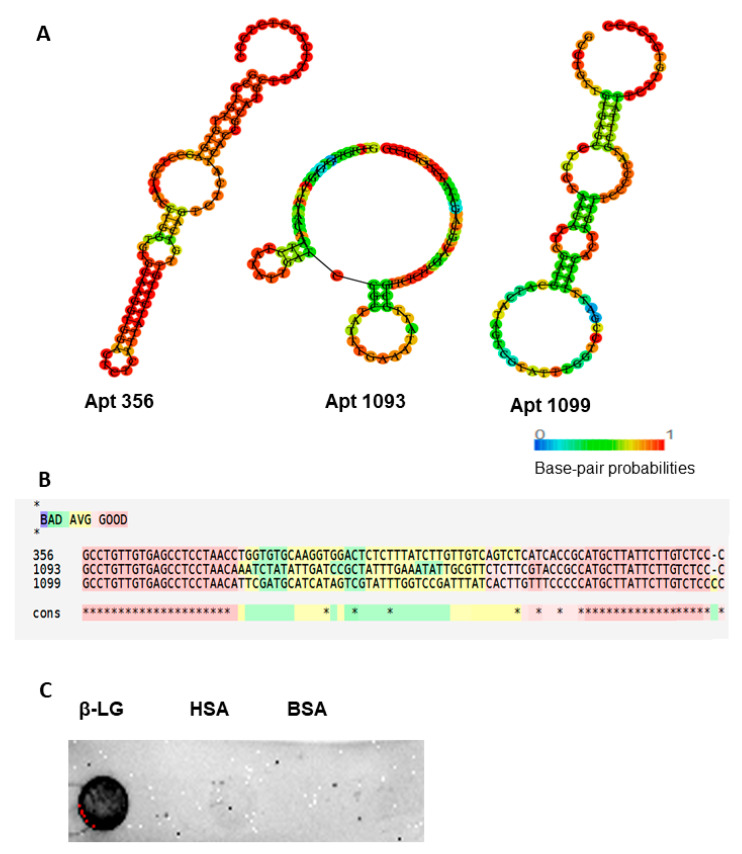
Analysis of the selected Apt 356. (**A**) The predicted secondary structure of Apt 356, generated using RNAfold. (**B**) Multiple sequence alignment of the selected aptamer sequences by T-Coffee. (**C**) 100 µg of target protein β-LG and negative controls BSA and HSA were incubated with 400 pmol biotinylated Apt 356, and the binding was analyzed by dot blot analysis. * means perfect conservation.

A comprehensive analysis of the NGS output (1142 sequences) was performed. Sequences 356, 1093, and 1099, with an increased similarity, were selected for the dot blot analysis (Supplementary Figure S1). The predicted secondary structure of the aptamer sequences, generated using RNAfold, and the multiple sequence alignment results obtained with the consensus meta-method M-Coffee (based on T-Coffee), are shown in Figure 2A,B. Dot blot analyses were performed to evaluate the binding of the biotinylated Apt 356 to the target protein β-LG (Figure 2C). Apt 356 demonstrated strong specific binding to the target protein β-LG, while no binding was observed to the negative controls BSA and HSA.

### 3.3. QCM Measurements

**Figure 3:**
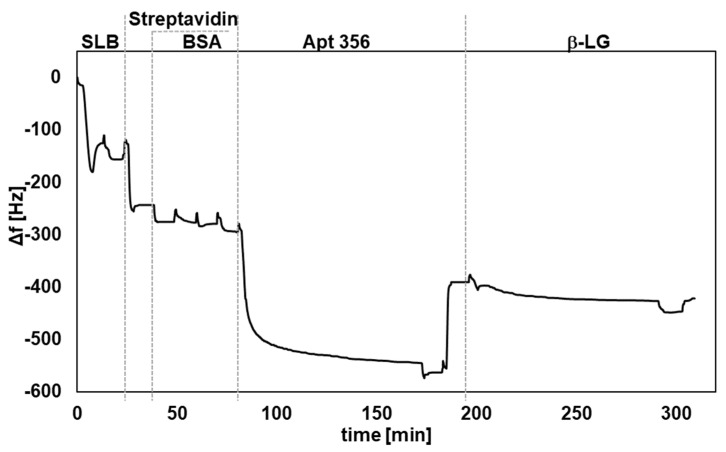
Selected QCM measurement for the real-time detection of β-LG. The frequency changes correspond to the different steps of surface modification and target binding (β-LG with a concentration of 75 µg/mL).

The QCM technology enables the real-time detection of molecular binding events and is widely used for a broad range of biochemical and medical applications. In this study, an aptasensor for the detection of β-LG was established. The β-LG-specific aptamer was immobilized on the sensor surface, serving as the recognition element. To mimic a physiologically relevant environment, an SLB was formed on the sensor and functionalized using streptavidin–biotin interactions to anchor the Apt 356 to the surface. Measurements were conducted at pH 3.5. The timeline of a typical QCM measurement is depicted in Figure 3.

**Figure 4:**
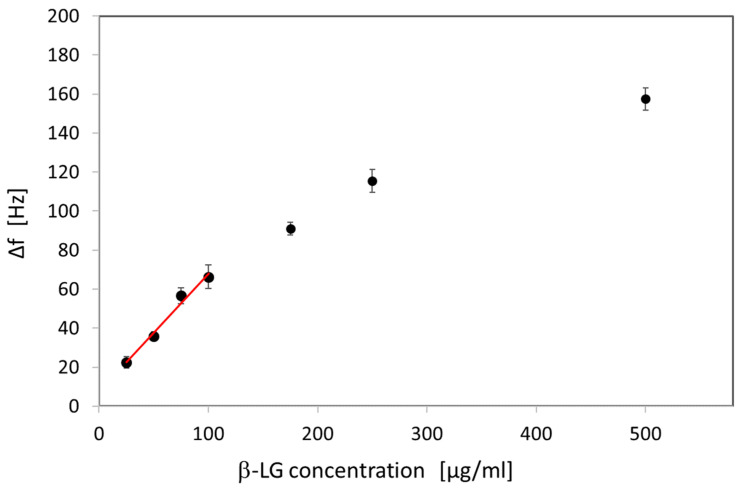
QCM measurements for different β-LG concentrations. To determine the LOD of the sensor system, the frequency shifts for β-LG concentrations of 25, 50, 75, 100, 175, 250, and 500 μg/mL were measured.

β-LG was applied in different concentrations to the aptasensor, ranging from 25 to 500 µg/mL. The lowest concentration of 25 µg/mL resulted in a frequency shift of 23 ± 3 Hz. Higher concentrations of 50, 75, 100, 175, 250, and 500 μg/mL showed frequency shifts of 36 ± 2 Hz, 57 ± 4 Hz, 66 ± 6 Hz, 91 ± 3 Hz, 115 ± 6 Hz, and 157 ± 6 Hz, respectively. The curve showed a linear relationship in the range of 25 to 100 µg/mL (R^2^ = 0.9824) with a slope of 607.8. Higher concentrations of β-LG led to saturation of the sensor surface with a reduced binding capacity for the target (Figure 4). The limit of detection (LOD) was calculated from the linear range as 0.04 µg/mL, corresponding to a concentration of 2.2 nM. These values are in good agreement with LODs reached with similar systems. So, Pan et al. [20] developed a QCM-based sensor with an anti-β-LG antibody as recognition element with the same LOD of 0.04 µg/mL.

## 4. Discussion

β-LG is the major whey protein and one of the principal allergens in bovine milk, making its accurate detection critical for food safety, especially for individuals with milk protein allergies. Even trace amounts of β-LG in processed or supposedly non-dairy foods can trigger severe allergic reactions. As such, reliable and rapid detection of β-LG is vital to ensure proper labeling, regulatory compliance, and consumer protection.

In this study, we successfully developed a novel aptamer-based biosensing platform for the detection of β-LG using the combinatorial chemistry method SELEX for the aptamer selection and QCM-D for real-time detection. Compared to current established analytical techniques such as ELISA [21] and liquid chromatography-mass spectrometry (LC-MS) [22], our aptamer-based detection using QCM technology offers a promising alternative.

Aptamers, which are short single-stranded nucleic acids with high specificity and affinity for target molecules, provide a reliable, rapid, and label-free approach for allergen detection. When combined with QCM, aptamer-based sensors enable real-time detection, making them a valuable tool for improving food safety and allergen monitoring. Furthermore, our previously developed surface functionalization for a QCM-D-based aptasensor [12] mimics an artificial cell membrane and thus creates a physiologically close environment for the binding of the target to the sensor.

Our QCM-D-based β-LG aptasensor system offers several advantages. Its real-time detection capability reduces operational complexity and eliminates the need for additional reagents, thereby lowering cost and analysis time. Furthermore, the use of aptamers instead of antibodies provides additional benefits, including improved thermal and chemical stability and batch reproducibility, making them more suitable for industrial applications than traditional methods [23,24].

The selection strategy employed in this study was deliberately designed to enhance the likelihood of selecting aptamers capable of binding to β-LG under physiologically relevant conditions. β-LG carries a net negative charge at pH 7.4, which typically poses a challenge for the selection of nucleic acid-based ligands, such as aptamers, due to the electrostatic repulsion between negatively charged backbones. To address this, we implemented a gradual pH increase during the selection rounds, with the rationale that this would favor the enrichment of aptamer candidates capable of binding to β-LG despite its negative charge at neutral pH.

This strategy proved effective, as confirmed by the dot blot assay performed at pH 7.4, where the selected aptamer demonstrated specific binding to β-LG. Importantly, no binding to the negative control proteins, HSA and BSA, was observed, although both are also negatively charged at this pH. The absence of cross-reactivity supports the specificity of the aptamer for β-LG and suggests that the aptamer recognizes unique structural or conformational features of β-LG, rather than relying solely on charge-based interactions.

Overall, the approach of gradually increasing the pH during selection appears to be a viable strategy for selecting aptamers that maintain specific target interactions under physiologically relevant conditions, and could be broadly applied in similar contexts where target proteins exhibit a net negative charge at a neutral pH.

The favorable outcomes achieved with the β-LG aptamer under controlled laboratory conditions provide a solid foundation for further development. Future studies will focus on validating the sensor’s performance across different pH conditions, and in both real and processed food matrices, thereby demonstrating its applicability in complex and practical settings. Moreover, systematic evaluation of the sensor’s reusability, long-term operational stability, and potential for integration into portable platforms will contribute significantly to its technological maturity and readiness for widespread implementation in food allergen monitoring systems.

## 5. Conclusions

In this study, an innovative aptasensor was developed for the detection of the milk allergen β-LG. By combining Apt 356, selected through membrane-based SELEX, with a QCM-D system functionalized with a lipid-mimicking membrane, the biosensor enabled rapid, label-free, and real-time detection of β-LG. While there have been some studies on aptasensors for β-LG protein, to the best of our knowledge, this is the first QCM-based aptasensor specifically designed for β-LG protein detection.

With further optimization and validation in complex food matrices, this biosensor could serve as a valuable tool for improving food safety, ensuring regulatory compliance, and ultimately protecting public health.

## Figures and Tables

**Table 1:** Conditions of the selection cycles.

SELEX Condition	Specifications
β-LG	20 µg for each round
Incubation time	1 h
Oligonucleotide amount	1st round: 2 nmol start library Following rounds: 200 pmol of the selected pool
pH	1–5 rounds: 4.3 6–9 rounds: 5.3 9 rounds: 6.3 10–12 rounds: 7.4
Washing steps	1 round: 1x washing buffer 2–5 rounds: 2x washing buffer 6–12 rounds: 3x washing buffer
Elution	2x with 7 M urea (95 °C for 10 min)
PCR cycles	1st round: 16 2–12 rounds: 8

## Data Availability

The datasets used and/or analyzed during the current study are available from the corresponding author upon reasonable request.

## Supplementary Materials

The following supporting information can be downloaded at https://www.mdpi.com/article/10.3390/bios15090563/s1, Figure S1: Binding analysis of different aptamers (356, 1093, and 1099) after the immobilization of 2 or 5 µg β-LG using dot blot assay.
