# Supplementary material for: Development of a QCM-D-Based Aptasensor for the Real-Time Detection of β-Lactoglobulin
Source: Biosensors (Basel). 2025 Aug 27;15(9):563. doi: 10.3390/bios15090563 (PMC12468009; doi:10.3390/bios15090563)
Supplement: Supplementary file 1 [file biosensors-15-00563-s001.zip › biosensors-3743814-supplementary.pdf]

Supplementary Data:

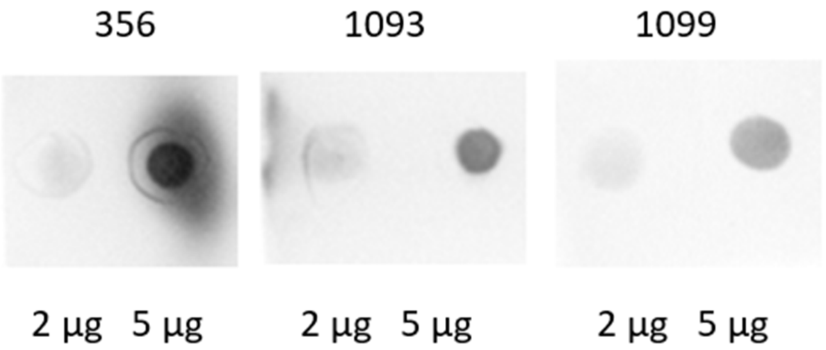

Figure S1: Binding analysis of different aptamers (356, 1093, and 1099) after the immobilization of 2 or 5 µg β-LG using dot blot assay.
